# Current progress in understanding the molecular pathogenesis of burn scar contracture

**DOI:** 10.1186/s41038-017-0080-1

**Published:** 2017-05-22

**Authors:** Jianglin Tan, Jun Wu

**Affiliations:** 1 Institute of Burn Research, State Key Laboratory of Trauma, Burns and Combined Injuries, Chongqing Key Laboratory for Disease Proteomics, Southwest Hospital, Third Military Medical University, Chongqing, 400038 China; 2 Department of Burns, First Affiliated Hospital of Sun Yat-sen University, Guangzhou, 510080 China

**Keywords:** Scar, Contracture, Burn, Molecular pathogenesis

## Abstract

Abnormal wound healing is likely to induce scar formation, leading to dysfunction, deformity, and psychological trauma in burn patients. Despite the advancement of medical care treatment, scar contracture in burn patients remains a challenge. Myofibroblasts play a key role in scar contracture. It has been demonstrated that myofibroblasts, as well as inflammatory cells, fibroblasts, endothelial cells, and epithelial cells, secrete transforming growth factor-β1 (TGF-β1) and other cytokines, which can promote persistent myofibroblast activation via a positive regulation loop. In addition to the cellular contribution, the microenvironments, including the mechanical tension and integrin family, are also involved in scar contracture. Most recently, eukaryotic initiation factor 6 (eIF6), an upstream regulator of TGF-β1, has been demonstrated to be involved in myofibroblast differentiation and contraction in both in vitro fibroblast-populated collagen lattice (FPCL) and in vivo external mechanical stretch models. Moreover, the data showed that P311 could induce the transdifferentiation of epidermal stem cells to myofibroblasts by upregulating TGF-β1 expression, which mediated myofibroblast contraction. In this review, we briefly described the most current progress on the biological function of myofibroblasts in scar contracture and subsequently summarized the molecular events that initiated contracture. This would help us better understand the molecular basis of scar contracture as well as to find a comprehensive strategy for preventing/managing scar contracture.

## Background

It is commonly accepted that scars are a pathologic wound healing response to burn injuries, traumatic injuries, and surgeries. Hypertrophic scars and keloids, which only occur in humans, present with exuberant scar formation [1]. Although these disorders do not pose a health risk, the scar contracture resulting in dysfunction and deformity remains a challenge in the clinic [2, 3]. The management of the scar contracture, such as surgical intervention, drugs, silicone materials, pressure therapy, splinting, lasers, and radiation, is used to control scar formation and contracture, but it is still far from achieving our expected outcomes [4]. Schneider found that 620 of the 1865 analyzed adult burn patients developed at least one joint contracture, which meant 33% of patients had dysfunction in their joints after burn injuries [5].

Wound healing proceeds through three overlapping stages. The inflammatory stage is triggered by injury, wherein platelets, neutrophils, and macrophages release inflammatory mediators and cytokines that participate in the recruitment of inflammatory cells, fibroblasts, endothelial cells, and epithelial cells. The proliferative stage involves fibroblast activation, myofibroblast differentiation, and extracellular matrix (ECM) deposition. In this phase, the myofibroblasts have acquired contractile properties that can contract the wound and promote re-epithelialization. The third healing stage is matrix remodeling, including scar tissue remodeling. During this stage, the persistent activation of myofibroblasts, imbalance of deposition and degradation of ECM, and poor arrangement of newly formed fibers can lead to scar formation.

Myofibroblasts, a type of cell differentiated from quiescent fibroblasts and other cells, have been demonstrated to play an essential role in the induction and maintenance of scar contracture. In normal acute wound healing, the myofibroblasts are temporally limited and cleared by apoptosis in the third healing phase when the tissues are repaired. However, in hypertrophic scars and keloids, these myofibroblasts persist at a high number for long periods and promote the synthesis of α-smooth muscle actin (α-SMA), transforming growth factor-β1 (TGF-β1), and other growth factors, and they have sustained contractile ability via the TGF-β1 positive loop [6].

## Review

### The origins and characteristics of myofibroblasts

In the inflammatory stage, fibrocytes and fibroblasts are believed to be activated in response to inflammatory factors; they then migrate to the location of injury based on a chemoattractant gradient and differentiate into myofibroblasts. In general, most myofibroblasts are derived from fibroblast differentiation around the local wound area [7]. In addition, other myofibroblasts may originate from pericytes [8], smooth muscle cells from the vasculature [9], fibrocytes from bone marrow-derived peripheral blood [10], epithelial cells through the epithelial-mesenchymal transition (EMT) [11], epidermal stem cells [12, 13], local mesenchymal stem cells, and bone marrow-derived mesenchymal stem cells [14].

**Fig. 1:**
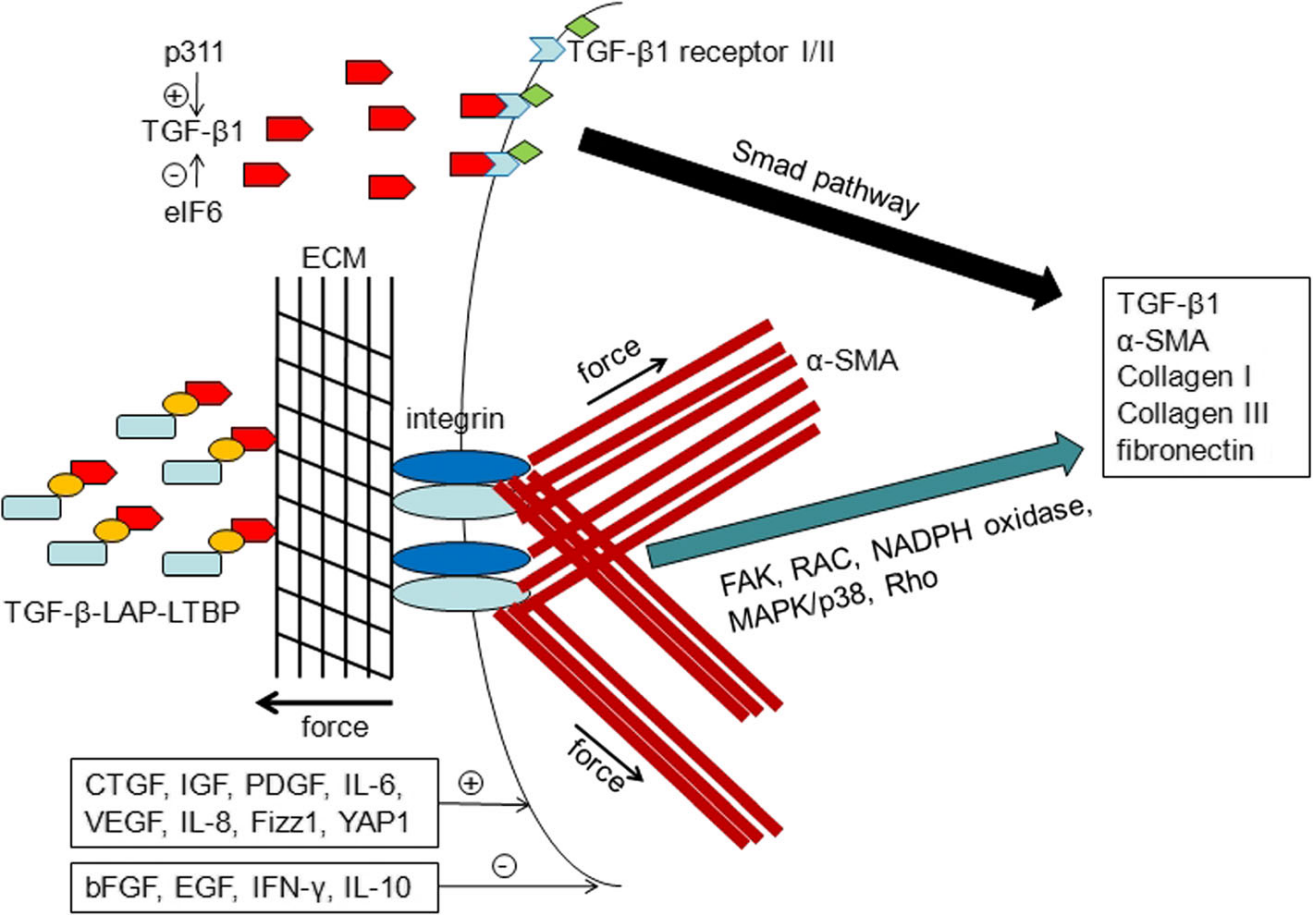
The cytokines and mechanical environment contribute to myofibroblast contraction: The inflammatory factors and growth factors such as TGF-β1, CTGF, IGF, PDGF, VEGF, IL-6, IL-8, Fizz1, and YAP1 could upregulate the expression of TGF-β1, α-SMA, collagen I, collagen III, and fibronectin via a positive feedback loop. The exogenous mechanical force can also promote the expression of α-SMA via FAK, RAC, NADPH oxidase, MAPK/p38, and Rho signaling pathways, enhancing the contractile force. bFGF, EGF, IFN-γ, and IL-10 can inhibit the myofibroblasts differentiation, thereby decreasing the contraction. P311 could upregulate the TGF-β1 expression. In contrast, eIF6 inhibits the TGF-β1 expression as an upstream regulator

The myofibroblast cell is a phenotypically intermediate cell type between fibroblasts and smooth muscle cells. The myofibroblasts exhibit the characteristics of smooth muscle cells, containing high-contractile stress fibers. The stress fibers consist of α-SMA protein, which can be used to differentiate between myofibroblasts and fibroblasts in tissues. However, it remains difficult and complicated to discriminate between myofibroblasts and other contractile cells, such as smooth muscle cells, pericytes, and myoepithelial cells, even if the smooth muscle cells express smooth muscle myosin heavy chain, h-caldesmon (H-CAD), smoothelin, and the muscle intermediate filament protein, desmin, which are absent from myofibroblasts [15]. There are a lot of cytokines and mechanical microenvironment factors that contribute to scar contracture (Fig. 1).

### The cytokine contribution to scar contracture

#### Transforming growth factor-β family

It has been demonstrated that TGF-β1 is one of the most important factors controlling myofibroblast differentiation and function. TGF-β is found in all tissues, and it consists of three isoforms, β1, β2, and β3. Binding of active TGF-β1 to the TGF-β receptor type II leads to the phosphorylation and recruitment of TGF-β receptor type I. The heteromeric receptor complex induces the phosphorylation of Smad2/3, followed by association with Smad4. Subsequently, the Smad complex translocates into the nucleus to enhance gene transcription via cooperation with DNA transcription factors. Additionally, it induces overproduction of α-SMA, collagen I, collagen III, fibronectin (FN), and other cytokines [16]. TGF-β1 can also reduce matrix metalloproteinases (MMPs) activity via decreasing proteases, such as tissue inhibitor of metalloproteinases (TIMPs) I and II [17]. The TGF-β/Smads signaling pathway is a positive autocrine loop in both hypertrophic scar and keloid formation, which then increases the stress fiber stabilization and stiffness of the microenvironment.

Importantly, TGF-β1 is an inducer of myofibroblast differentiation, which is considered a potential therapeutic target for hypertrophic scars and keloids. It has been reported that peroxisome proliferator-activated receptor-γ (PPARγ) ligands, 15d-PGJ2 and GW7845, could inhibit the expression and phosphorylation of TGF-β1/Smads [18]. Either disruption or neutralization of TGF-β/Smads signaling by botulinum toxin type A, tetrandrine, baicalein, loureirin B, or the Uighur medicine ASMq can decrease the myofibroblast properties [19]. In addition, TGF-β1 could also promote myofibroblast differentiation independent of Smads signaling and instead act via the wnt, p38, and PI3K/PKA2 signaling pathways [20]. P311, identified by suppressive subtraction hybridization as potentially involved in smooth muscle (SM) myogenesis, was highly expressed in hypertrophic scars and could induce a TGF-β1-independent, nonfibrogenic myofibroblast phenotype [21, 22]. Furthermore, in the renal fibrosis model, it was found that overexpression of P311 was concurrent with the expression of α-SMA and TGF-β1 via the TGF-β1/Smad signaling pathway [23]. Eukaryotic initiation factor 6 (eIF6), acting as a key binding protein of P311 [24], has recently been demonstrated by our team as a novel upstream regulator to inhibit myofibroblast differentiation at the TGF-β1 transcription level via H2A.Z occupancy and Sp1 recruitment. Additionally, there is downregulation of α-SMA and collagen I expression [25]. In addition, our next study demonstrated that eIF6-mediated TGF-β1 can also be regulated by external mechanical stretch [26].

#### Positive growth factors and cytokines

In addition, many other growth factors show positive roles in myofibroblast differentiation, such as connective tissue growth factor (CTGF), platelet-derived growth factor (PDGF), insulin growth factor (IGF), and vascular endothelial growth factor (VEGF) [27, 28]. CTGF could synergize the action of TGF-β, promoting ECM production and scar contracture. Inhibition of the expression of CTGF can reduce the formation of hypertrophic scars. Likewise, PDGF is released into the wound and induces myofibroblast activation directly or in synergy with TGF-β1. PDGF stimulates fibroblast proliferation and regulates collagen synthesis via extracellular signaling-regulated kinase (ERK) and the PI3K/JNK signaling pathway. Blocking of PDGF receptors α and β was shown to inhibit myofibroblast formation. IGF acts as a mitogenic factor to enhance the expression of collagens I and III while also reducing the release of collagenase. VEGF could induce the expression of collagen I and promote scar formation [27].

Moreover, interleukin-6 (IL-6) and interleukin-8 (IL-8) increase α-SMA transcription in fibroblast cultures [29, 30]. Similarly, nerve growth factor enhanced α-SMA expression in fibroblasts [31]. Fizz1 induced the expression of α-SMA [32]. YAP1 contributes to the maintenance of a synthetic and contractile phenotype in fibrosis [33]. Agonists of myofibroblast contraction, such as angiotensin-II, endothelin-1, and thrombin, can upregulate the expression of α-SMA [34–36]. As a result, these inflammatory cytokines play roles in myofibroblast differentiation and scar contracture [37].

#### Negative growth factors and cytokines

In contrast, some negative factors act against myofibroblast differentiation. Basic fibroblast growth factor (bFGF, FGF2), epidermal growth factor (EGF), interferon-γ (IFN-γ), interleukin-10 (IL-10), prostaglandin E2 (PGE2), eIF6, and TGF-β3 have been shown to suppress the expression of α-SMA and ECM synthesis [25, 38, 39]. bFGF has been shown to suppress myofibroblast function and α-SMA expression by antagonizing TGF-β1. EGF could negatively regulate the role of TGF-β1 in inducing myofibroblast contraction through attenuating autologous release of TGF-β1. IFN-γ and IL-1β induced apoptosis in myofibroblasts and antagonized TGF-β1 regulation and production [40, 41]. Additionally, TGF-β3 exerted suppressive effects on myofibroblasts in a 3-D repair model [42].

### The mechanical microenvironment contribution to scar contracture

#### Mechanical tension

Hypertrophic scars frequently occur at particular sites, including the anterior chest wall, auricle, scapula, and suprapubic regions, which are frequently subjected to the high stretch tension from the natural daily movements of the body [43]. The activity of myofibroblasts depends on the mechanical microenvironment. The stress fibers, fibronectin, and smooth muscle actin appear earlier in the inflammatory stage, which can increase the mechanical tension by association with the ECM. With mechano-sensitive ion channels in the plasma membrane, integrin-mediated stress perception and geometrical changes of myofibroblasts can sense stress [44]. Fibrosis tissue exerts greater forces of 20–100 kPa with a collagen-dense tendon [45]. In contractile wound granulation tissue and myofibroblasts cultured on elastic substrates, the threshold stiffness for the expression of α-SMA in stress fibers is approximately 20 kPa [46]. In liver fibrosis, hepatic stellate cells can be activated with 15 kPa of pressure and then differentiate into α-SMA-positive myofibroblasts [47].

α-SMA has been demonstrated as a mechano-sensitive protein that induces a rapid mechanism to control the myofibroblast contractile function. There is positive regulation between stress and α-SMA expression level. The exogenous mechanical force, when applied through integrins, activates the Rho or mitogen-activated protein kinase (MAPK)/p38 signaling pathway, which then enhances the activation of serum response factor (SRF) and increases α-SMA transcription and incorporation into actin filaments. The persistent upregulation of α-SMA increases the intracellular tension and induces a higher force compared to the cytoplasmic actin stress fiber, which stimulates ECM contraction [48]. A decrease in the intracellular stress will make the myofibroblasts insensitive to external mechanical factors via interfering with α-SMA, inhibiting the Rho/Rho-associated kinase pathway effect on myosin activity, interfering with mega karyoblastic leukemia factor 1 (MKL1) that is linked with mechanical stress, and interfering with YAP/TAZ transcription factors that mediate mechano-responses [49–52].

#### Integrin family

Integrin is an essential mechano transducer that is connected with stress fibers in cells and the ECM surrounding the cells [53]. These signal mediators are cell surface receptors that consist of two isoforms, α and β subunits. Integrin is involved in the activation of latent TGF-β1 and production of collagen, α-SMA and connective tissue growth factor (CTGF) via reactive FAK, RAC, and NADPH oxidase as well as an oxygen species (ROS)-dependent mechanism [54]. Fibroblasts with integrin β_1_ knockout are less able to adhere to and contract the ECM [55]. Integrins α_1_β_1_, α_2_β_1_, α_3_β_1_, α_v_β_5_, α_5_β_1_, α_v_β_3_, α_v_β_6_, α_v_β_8_, and α_11_β_1_ were demonstrated to partially participate in fibroblast proliferation, collagen contraction, and myofibroblast differentiation [56]. Deletion of integrin α_3_β_1_ decreased the accumulation of myofibroblasts and collagen I, which decreased the fibrosis [57]. Moreover, some other integrins, such as integrins α_v_β_5_, α_v_β_3_, and α_8_β_1_, were shown to bind to LAP-TGF-β1 and are involved in TGF-β1 activation [58, 59].

## Conclusions

After injury, the quiescent fibroblasts and other original cells are activated in response to inflammatory signals, such as TGF-β1. Following the TGF-β/Smad signaling cascade, TGF-β1 enhances gene transcription, as demonstrated by the upregulation of α-SMA, collagen I/III, and other fibrotic genes. Meanwhile, the expression of TGF-β1 is also increased by a positive feedback loop. Furthermore, eIF6 and P311 were involved in myofibroblast differentiation and contraction via regulating the TGF-β1 expression. This indicated that eIF6 and P311 may be new potential target genes for treating scar contracture. In addition, there are other cytokines, such as growth factors and inflammatory relative factors, which can up/downregulate myofibroblast contracture. Continuing ECM alignment creates larger surfaces for adhesion formation, which connects TGF-β-LAP-LTBP and integrins. The larger adhesions permit the development of stronger stress fibers and generation of a higher contractile force.

In the wound repair phase, contraction can close the original wound and reduce the surface area. However, the continuation of contraction after wound healing can have high clinical morbidity of the joint contractures, functional loss, delayed return to work, and poor cosmetic results. The molecular basis/pathogenesis of scar contracture is complicated and includes cellular factors and environmental contributions. No single treatment method has been demonstrated to be effective [60]. To find the appropriate treatment for scar contractures, we prefer to consider a comprehensive strategy, including cytokines and environmental aspects, and then translate the basic discoveries into potential therapies.

## Abbreviations

bFGF: Basic fibroblast growth factor
CTGF: Connective tissue growth factor
EGF: Epidermal growth factor
eIF6: Eukaryotic initiation factor 6
EMT: Epithelial-mesenchymal transition
ERK: Extracellular signaling-regulated kinase
FN: fibronectin
FPCL: Fibroblast-populated collagen lattice
H-CAD: h-Caldesmon
IGF: Insulin growth factor
MAPK: Mitogen-activated protein kinase
MKL: Megakaryoblastic leukemia factor
MMP: Matrix metalloproteinase
PDGF: Platelet-derived growth factor
PG: Prostaglandin
PPARγ: Peroxisome proliferator-activated receptor-γ
SRF: Serum response factor
TGF-β1: Transforming growth factor-β1
TIMPs: Tissue inhibitor of metalloproteinases
VEGF: Vascular endothelial growth factor
α-SMA: α-Smooth muscle actin
